# Chromosomal and Plasmid-Encoded Factors of *Shigella flexneri* Induce Secretogenic Activity *Ex Vivo*

**DOI:** 10.1371/journal.pone.0049980

**Published:** 2012-11-16

**Authors:** Christina Faherty, Jill M. Harper, Terez Shea-Donohue, Eileen M. Barry, James B. Kaper, Alessio Fasano, James P. Nataro

**Affiliations:** 1 Department of Medicine, Center for Vaccine Development, University of Maryland School of Medicine, Baltimore, Maryland, United States of America; 2 Mucosal Biology Research Center, University of Maryland School of Medicine, Baltimore, Maryland, United States of America; 3 Department of Microbiology and Immunology, University of Maryland School of Medicine, Baltimore, Maryland, United States of America; Wadsworth Center, New York State Dept. Health, United States of America

## Abstract

*Shigella flexneri* is a Gram-negative, facultative intracellular pathogen that causes millions of cases of watery or bloody diarrhea annually, resulting in significant global mortality. Watery diarrhea is thought to arise in the jejunum, and subsequent bloody diarrhea occurs as a result of invasion of the colonic epithelium. Previous literature has demonstrated that *Shigella* encodes enterotoxins, both chromosomally and on the 220 kilobase virulence plasmid. The *Shigella* Enterotoxins 1 and 2 (ShET1 and ShET2) have been shown to increase water accumulation in the rabbit ileal loop model. In addition, these toxins increase the short circuit current in rabbit tissue mounted in Ussing chambers, which is a model for the ion exchange that occurs during watery diarrhea. In this study, we sought to validate the use of mouse jejunum in Ussing chamber as an alternative, more versatile model to study bacterial pathogenesis. In the process, we also identified enterotoxins in addition to ShET1 and ShET2 encoded by *S. flexneri*. Through analysis of proteins secreted from wildtype bacteria and various deletion mutants, we have identified four factors responsible for enterotoxin activity: ShET1 and Pic, which are encoded on the chromosome; ShET2 (encoded by *sen* or *ospD3*), which requires the type-III secretion system for secretion; and SepA, an additional factor encoded on the virulence plasmid. The use of mouse jejunum serves as a reliable and reproducible model to identify the enterotoxins elaborated by enteric bacteria. Moreover, the identification of all *Shigella* proteins responsible for enterotoxin activity is vital to our understanding of *Shigella* pathogenicity and to our success in developing safe and effective vaccine candidates.

## Introduction

Enteric infections resulting in diarrhea are prevalent throughout the developed and developing world. Children with frequent diarrheal episodes are at risk for life-long physical and mental defects [1]. While viruses and parasites cause a significant amount of diarrheal disease, bacterial pathogens, including the pathogenic strains of *Escherichia coli*, *Shigella,* and *Salmonella*, are a leading cause of diarrheal disease in the developing world [2]. Preventive and prophylactic measures have been difficult to develop and implement, making diarrheagenic bacteria a nuisance to public health and detrimental to the health of the affected populations. The lack of available animal models has been a hindrance to the process of vaccine and drug development.

*Shigella* species comprise a leading cause of diarrhea-associated morbidity and mortality in children under the age of five years in developing countries [3]. This age group is particularly susceptible to the effects of acute and chronic infections, although *Shigella* is clinically important in all age groups in the developing world. In addition to dehydration and the inability to absorb nutrients, children who are continuously exposed to pathogenic bacteria suffer from both physical and developmental milestone delays [1]. Stools from these children frequently contain multiple infectious organisms; however, *Shigella flexneri* 2a is the most prominent bacterial pathogen isolated from children suffering from diarrhea between the ages of 2 and 5 years [3]. Although the invasion process has been thoroughly dissected, the mechanisms by which *Shigella* induces secretory diarrhea are less defined. Like many other pathogenic bacteria, *S. flexneri* 2a possesses a type-III secretion system (T3SS) that produces several proteins required for invasion of intestinal cells. At least one T3SS effector has been identified as a putative toxin [4], [5]. Most, if not all, pathogenic bacteria elaborate one or more toxins during the course of infection. These toxins have diverse activities that include, but are not limited to, colonization, proteolytic activity, cellular toxicity, and increasing intestinal secretion [6], [7].

To date few studies have addressed the effects of extracellularly secreted bacterial products during *S. flexneri* 2a infection. Studies performed in the 1990s described two toxins produced by *S. flexneri* 2a, *Shigella* enterotoxin 1 (ShET1) and *Shigella* enterotoxin 2 (ShET2), that contribute to secretory activity in rabbit intestine *in vivo* and *in vitro* [4], [8], [9]. An epidemiological study of 383 *Shigella* isolates found that ShET1 was present in 30% of all *Shigella* isolates while ShET2 was present in 80% of isolates. Watery diarrhea is present in almost all shigellosis cases, and typically proceeds to dysentery. In fact in mild infections, watery diarrhea is the only clinical manifestation. [10], [11]. ShET1 is encoded on the chromosome in the *she* pathogenicity island (PAI), and is only found in *S. flexneri* [8]. ShET2 is a T3SS effector encoded on the 220 kb invasion plasmid [5]. Previous studies reported the ability of bacterial supernatants prepared in iron-depleted medium to increase secretion in both Ussing chambers and the rabbit ileal loop model [4], [8], [9]. It was theorized that the secretion of ShET1 and ShET2 in the upper intestine (*e.g.* jejunum) would increase fluid secretion which, in turn, would facilitate the transit of bacteria to the colon, the site of invasion. This observation accounts for the watery diarrhea, as opposed to the scant bloody diarrhea, that occurs as a result of PMN infiltration at the site of invasion [12].

The overarching goal of this study was to validate mouse tissue as a useful model of intestinal secretion caused by exposure to secreted toxins. We also aimed at identifying all secreted factors that contribute to jejunal secretion to better define the mechanism of *Shigella*-induced diarrhea. *In vivo* intestinal secretion as a result of *S. flexneri* infection is difficult to study as the pathogen will only infect non-human primates at the natural site of infection [13]. Ussing chambers are a well-established system to define secretion from intestinal tissue *ex vivo*, and provide an alternative to *in vivo* studies [14]. Previous studies defined the response of rabbit jejunum to secreted *Shigella* toxins and recombinant proteins in the Ussing chambers [4], [8], [9]. We extended this secretory model to include mouse jejunum, which has proven to respond similarly to rabbit tissue. Herein we demonstrate the utility of mouse intestine as an affordable and informative model for defining secretory activity of bacterial toxins. Our study implicates both chromosomal and plasmid-encoded toxins as contributors to intestinal secretion caused by *S. flexneri* 2a. Identification of all of the enterotoxins encoded by *S. flexneri* is vital to our understanding of *Shigella* pathogenesis and may lead to targets suitable for drug and vaccine development.

## Materials and Methods

### Strains and Growth Conditions

Table 1 lists the bacterial strains and plasmids used in this study. Table 2 lists the enterotoxins expressed for each bacterial strain used in this study. Bacteria were routinely cultured at 37°C either in Luria-Bertani broth with aeration, or on tryptic soy broth plates with 1.5% agar and 0.025% Congo Red (CR; Sigma). Antibiotics were used at the following concentrations: kanamycin, 50 µg/ml; streptomycin, 50 µg/ml; and chloramphenicol, 5 µg/ml. Guanine supplementation (0.005%, Sigma) was used for strains harboring the Δ*guaBA* mutations (strain 1208S).

**Table 1:** Strains and plasmids used in this study.

Name	Description	Reference
**Strains**		
2457T	Wildtype *S. flexneri* 2a	[36]
BS103	Plasmid-cured 2457T	[37]
BS652	2457T/*spa47::aadA*	[38]
1377	2457T/*pic/set1*AB*::aph-3*	[16]
1380	1377/type-III secretion system mutant	This study
1522	1377/*pic^+^/set1*AB*^+^*	[16]
1542	1377/*pic^+^*	[16]
BS796	2457T/*ospD3::cat*	ATM; this study
1208S	2457T/Δ*guaBA*+Δ*set1*AB+Δ*ospD3*	[39]
	2457T/Δ*sepA*	BAM
*E. coli* HS	Commensal *E. coli*; NaI^r^	[23]
**Mutant Construction**		
Strains		
BS766	2457T transformed with pKM208	[38]
Plasmids		
pKD3	oriR6K, *bla*, *cat*	[15]
pKM208	Temperature-sensitive *red-, gam-, lacI-*expressing plasmid driven by *P_Tac_* promoter, *bla*	[15]

**Table 2:** Toxins expressed in the *Shigella flexneri* strains analyzed.

Strain	ShET1	Pic	ShET2	SepA
2457T	+	+	+	+
BS103	+	+	−	−
BS652	+	+	−	+
1377	−	−	+	+
1380	−	−	−	+
1522	+	+	+	+
1542	−	+	+	+
BS796	+	+	−	+
1208S	−	−	−	+
Δ*sepA*	+	+	+	−
*E. coli* HS	−	−	−	−

### Mutant Construction

Strain 1377 (Δ*pic* and Δ*set1*AB) was constructed using a modification of the λ red linear recombination method [15] in which the entire *pic/set1*AB region was replaced with a kanamycin/sacB cassette. Selection for the mutation was performed using kanamycin. Sucrose sensitivity and PCR analysis verified that the mutation was achieved at the appropriate site. Strain 1380 is a spontaneous T3SS mutant, and was selected for by red/white colony screenings on CR plates. To construct 1522, the kanamycin/sacB cassette from 1377 was removed by a second linear recombination step in which the wildtype *pic/set1*AB locus was replaced back into the region of interest [16]. To construct 1542 the *pic/set1*AB locus with four point mutations to terminate transcription of *set1*A and *set1*B while maintaining transcription of *pic* was replaced back into the region of interest [16]. 1522 and 1542 were selected via sucrose resistance and screened for kanamycin sensitivity. DNA sequencing confirmed restoration of the wildtype or point mutation locus.

Strain BS796 (Δ*ospD3*) was constructed using the λ red linear recombination method as previously described [15]. PCR was used to amplify a chloramphenicol resistance cassette gene (cat) from pKD3 (Table 1) which had 5′ and 3′ overhangs identical to the 5′ and 3′ regions of *ospD3*. The sequence of the forward primer was 5′ – TGA AAG GAA TAT ATA CAT ATG CCA TCA GTA AAT TTA ATC CCA TCA AGG CAT ATG AAT ACC TCC TTA GTT CC –3′ and the sequence of the reverse primer was 5′ – ATC ACC ACG AGA TAA TAT TCA GCT TTT TAT ATT CTT CAT AAT TT CTA GTG TGT AGG CTG GAG CTG CTT C –3′. Chloramphenicol resistant recombinants were purified and screened via PCR using primers that annealed to unique regions upstream and downstream of *ospD3* to detect the size difference due to the insertion of the chloramphenicol cassette. For this PCR reaction, the forward primer sequence was 5′ – GCC ATC AAT CAT CCA AAG GG –3′ and the reverse primer sequence was 5′ – CGG AGG TAA CAG ACC AGA CG –3′. This mutant, in the BS766 background, was used as the donor strain for transduction of 2457T (the recipient) using P1L4 [17], and selection for chloramphenicol resistance generated BS796. The mutation was verified by PCR.

### Animals Used in this Study

Female New Zealand White (NZW) rabbits were purchased from Charles River. Rabbits weighing between 2.3 and 3 kg were used in Ussing chamber experiments. Female C57BL/6 mice were purchased from the National Cancer Institute (Frederick, MD) and were used for Ussing experiments between 6 and 14 weeks of age.

### Preparation of Bacterial Supernatants

Samples were prepared as follows for use in Ussing studies. Overnight cultures of bacteria were subcultured into 600 ml Luria-Bertani Broth (LB) with the appropriate antibiotic selection or nutrient supplementation required for each strain of *S. flexneri* (see strains and growth conditions). Cultures were grown at 37°C with shaking until the optical density (OD_600_) reached 1.0. The bacteria were then pelleted by centrifugation at 5,000 rpm for 10 minutes at 4°C. Culture supernatants were subsequently filter-sterilized with a 0.45 µm vacuum filtration unit (Millipore) and stored at −20°C. The stored supernatants were thawed at 4°C and concentrated using the Centricon Plus-70 centrifugal filter devices with a molecular weight cutoff of 30 kD (Millipore), according to manufacturer’s instructions. Concentrated proteins (averaging 30 µg/µl) were resuspended in 1X PBS to a final volume of 2 ml, and samples were stored at −80°C in 500 µl aliquots.

### Preparation of Iron-depleted LB

In order to deplete the iron from LB, fifty grams of Chelex-100 resin (Bio-Rad) was added to 1 L of LB according to manufacturer’s instructions and stirred for 2 hours, after which the solution was filtered through a 0.45 µM vacuum filter unit with receiver bottle (Millipore). MgSO_4_ was added to a final concentration of 2 mM and CaCl_2_ was added to a final concentration of 0.1 mM. All solutions were prepared with iron-free water in iron-free containers. The pH of the solution was adjusted to 7.4 with concentrated HCl. The iron-free LB was then filtered a second time through a 0.45 µM filter to ensure sterility. Medium was used immediately or stored at 4°C until use.

### Ussing Chamber Studies

Assessment of secretory activity in response to secreted bacterial proteins was performed using jejunum mucosae stripped of muscle and mounted in Ussing chambers [18]. For rabbit jejunum 0.5206 cm^2^ tissue was exposed for analysis in the Ussing chambers, while 0.1256 cm^2^ tissue was exposed for mouse jejunum. While in the Ussing chambers, the tissue was short circuited every 50 s at 1 V (World Precision Instruments DVC, 1000 voltage clamp) and the short circuit current (Isc) was monitored continuously. The potential difference was measured using agar-salt bridges and electrodes. After equilibration of 0.126 cm^2^ of tissue in 10 ml of Krebs’ buffer (118 mM NaCl, 4.7 mM KCl, 1.2 mM KH2PO4, 1.2 mM MgSO4, 4.2 mM NaHCO3, 2 mM CaCl2, 10 mM glucose, 200 mM sulphinpyrazone and 10 mM Hepes, pH 7.4), a test for glucose absorption was conducted to assess the responsiveness of the tissue. To test the function of the sodium linked glucose transporter and, therefore, the integrity of the tissue, 20 mM glucose was added to the mucosal chamber while an iso-osmotic solution of mannitol was added to the serosal chamber; and Isc determined. The tissues were washed and allowed to equilibrate once more, after which 400 µl of concentrated secreted *Shigella* proteins were added to the mucosal chamber and 400 µl of PBS was added to the serosal chamber. Isc was measured for at least 30 minutes post-toxin addition; the change in Isc (ΔIsc) reflects the maximum change during this time course. Responses from two tissue segments exposed to secreted proteins from one mouse were averaged to yield a mean response per animal. Each sample was tested on a minimum of four animals. For every experiment, secreted proteins from wildtype 2457T served as a positive control. Values are expressed as mean ± standard error of the mean (SEM).

### Congo Red Secretion Assay

The Congo red (CR) secretion assay was used to identify proteins secreted by the bacteria through the T3SS, and was performed as previously described [19]. Briefly, bacteria were grown to mid-log phase, resuspended in 1X PBS, and CR was added to a final concentration of 30 µg/ml. The bacteria were incubated at 37°C for 1 hour. After incubation, the bacteria were pelleted by centrifugation, and the supernatant was collected and filtered through a 0.22-µm-pore filter and stored at –20°C. The proteins in the supernatant represent the secreted proteins and were concentrated by trichloroacetic acid (TCA) precipitation. TCA pellets were resuspended in 50 µl sodium dodecyl sulfate (SDS) loading buffer for protein analysis and stored at –20°C. The bacterial pellets, representing the non-secreted proteins, were resuspended in 500 µl of SDS loading buffer and stored at −20°C.

### Protein and Western Blot Analysis

For total protein analysis each sample was resolved by SDS-polyacrylamide gel electrophoresis (PAGE), and Coomassie blue stain was used to visualize total protein. For Western blot analysis proteins were transferred to a PVDF membrane and blocked with 10% dry milk in 1X PBS. IpaB was detected with mouse monoclonal anti-IpaB antibody (1∶20,000 dilution) [20]. After washing, Alexa Fluor 700 goat anti-mouse immunoglobulin G (H+L) antibody (Molecular Probes) was added at a 1∶3,000 concentration in PBS-Tween with 10% dry milk for 1 h. All washes of the PVDF membrane were performed with 1X PBST for 5 minutes at room temperature. Protein gels and Western blots were scanned using the Odyssey infrared detection system (Li-Cor).

### Statistical Analysis

Two-tailed Student’s t-tests or analysis of variance (ANOVA) were used to assess significance with p<0.05 considered to be significant.

### Ethics Statement

This study was carried out in strict accordance with the recommendations in the Guide for the Care and Use of Laboratory Animals of the National Institutes of Health. The protocol was approved by the Institutional Animal Care and Use Committee (IACUC) at the University of Maryland at Baltimore (protocol #0111044). All efforts were made to minimize suffering during euthanasia of the mice.

## Results

### Iron-depletion of Medium does not Affect Enterotoxic Activity

**Figure 1:**
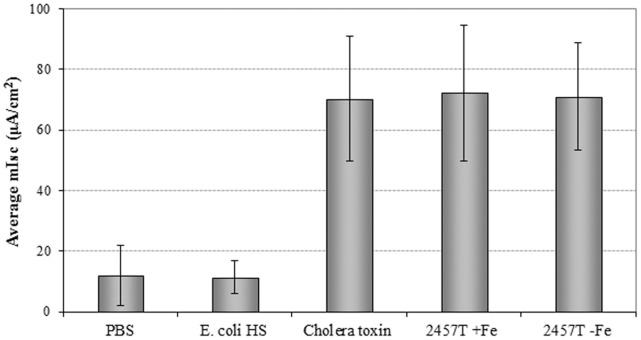
Iron depletion is not required for *S. flexneri* enterotoxin activity. The average change in short circuit current (µA/cm2) is plotted +/− the standard error for wildtype *S. flexneri* 2a strain 2457T with iron (+Fe) and without iron (-Fe). There is no significant difference between the two conditions. PBS and supernatant from *E. coli* HS served as negative controls while 40 µg of *Vibrio cholerae* toxin served as a positive control for inducing a change in Isc.

Previous studies with enteroinvasive *E. coli* required iron depletion in order to detect an enterotoxin effect in the rabbit ileal loop model and increases in Isc in the Ussing chambers [21]. These iron-depleted conditions were continued with studies for *S. flexneri*; however, it was never determined if iron-depletion was required to induce enterotoxin expression in *S. flexneri*. In this study, we tested whether enterotoxic activity of *S. flexneri* 2457T was only apparent after preparation with iron-depleted media. Cultures were prepared with and without Chelex-100 resin, and the culture supernatants were processed as described in the Materials and Methods. Supernatants from 2457T cultures prepared with and without the iron chelator both resulted in a significant increase in Isc, and no significant difference was detected between the two conditions on rabbit tissue (Figure 1). The *Vibrio cholerae* toxin served as the positive control [22] whereas PBS and supernatants derived from the commensal *E. coli* HS strain, which does not produce any enterotoxic activity [23], were employed as the negative controls. These observations were confirmed in experiments with mouse tissue (data not shown). As a result of these experiments, we maintained normal growth conditions, without iron depletion, for analysis of the *S. flexneri* deletion mutants.

### The Plasmid-cured Strain and a Type-III Secretion System Mutant Identify Loci of Putative Enterotoxin Genes

**Figure 2:**
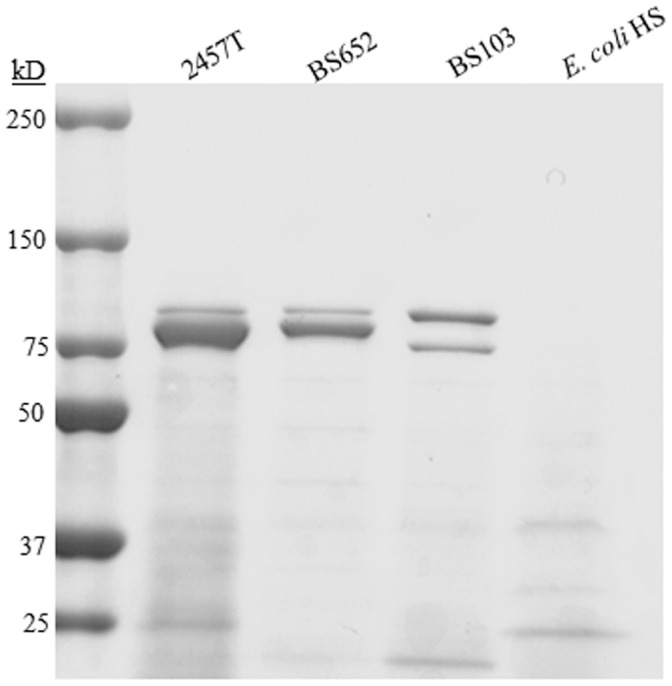
Coomassie-stained protein gel of the secreted proteins obtained from each strain of bacteria after growth and concentration of cultural supernatants. Wildtype *S. flexneri* 2a strain 2457T secretes the autotransporter proteins localized around 100 kD in addition to other type-III secretion system effector proteins at lower molecular weights. Strain BS652 has a defective T3SS but is still able to secrete the autotransporter SepA that is encoded on the virulence plasmid. Strain BS103 is the virulence plasmid-cured and therefore secrets only the chromosomal autotransporters. The negative control *E. coli* HS commensal strain secretes a minimal amount of protein.

In order to corroborate and expand previous studies on the location (chromosome, virulence plasmid, or both) of genes encoding enterotoxic factors, we compared the plasmid-cured strain of *S. flexneri* (strain BS103) and a T3SS mutant (strain BS652) to wildtype 2457T. BS652 harbors a deletion in *spa47*, which encodes the ATPase that drives the T3SS. This mutation results in assembly of the T3SS apparatus but prevents secretion of T3SS effector proteins [24], [25]. Therefore, proteins encoded on the virulence plasmid that do not require the T3SS for secretion are still present in the culture supernatants. *E. coli* HS served as a negative control for toxigenic activity. The protein profile of the concentrated supernatants for each strain is provided in Figure 2. High molecular weight proteins are encoded on both the chromosome and virulence plasmid, and represent the large autotransporter proteins encoded by the bacteria [26], [27], [28], [29]. The smaller proteins present in the 2457T supernatants represent the effector proteins secreted by the T3SS [30].

**Figure 3:**
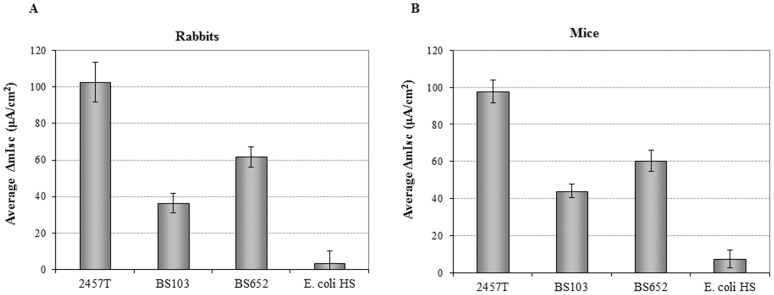
The *S. flexneri* chromosome and virulence plasmid encode enterotoxins that increase Isc in both rabbit and mouse tissue. The average change in short circuit current (mΔIsc, µA/cm^2^) is plotted +/− the standard error for wildtype *S. flexneri* strain 2457T, the virulence plasmid-cured strain BS103, and the Δ*spa47* mutant strain BS652. Analysis of BS103 and BS652 revealed that both the chromosome and virulence plasmid encode enterotoxins. *E. coli* HS served as a negative control. Experiments were performed with intestinal tissue from rabbits (A) and mice (B). All differences are significant, with *p*-values <0.01.

Analysis of the concentrated supernatants on rabbit tissue in the Ussing chambers demonstrated that 2457T consistently induced an increase in Isc of approximately 100 µA/cm^2^ while the enterotoxic signal triggered by strain BS103 was significantly lower compared to 2457T, with a consistent Isc of approximately 40 µA/cm^2^ (Figure 3A). The data suggested that the chromosome harbors genes encoding enterotoxins. The ΔIsc of approximately 60 µA/cm^2^ caused by strain BS652 was significantly lower than that induced by 2457T supernatants, but was also significantly higher than BS103 (Figure 3A). Since BS652 has a defective T3SS, the data indicated that there are two types of toxin contributions from the virulence plasmid. The first type is an effector secreted through the T3SS and the second type does not require the T3SS to be secreted. Based on the annotated sequence of the virulence plasmid and the appearance of supernatants on protein gel electrophoresis, we hypothesized that a high molecular weight autotransporter encoded on the plasmid would contribute to enterotoxin activity (see below). To develop a more practical and efficient *ex vivo* assay, we also performed analysis of the same strains on tissue isolated from mice. The supernatants were prepared and applied to the mouse jejunal tissue in the same manner as described for the rabbit jejunum. We found that 2457T, BS103, and BS652 induced similar changes in Isc in the mouse tissue as in the rabbit tissue (Figure 3B). Given the benefits of utilizing mouse tissue, we proceeded with mouse jejunum for the remainder of the experiments with 2457T supernatants serving as the positive control.

### Identification of the Genes Encoding the Chromosomal Enterotoxin

**Figure 4:**
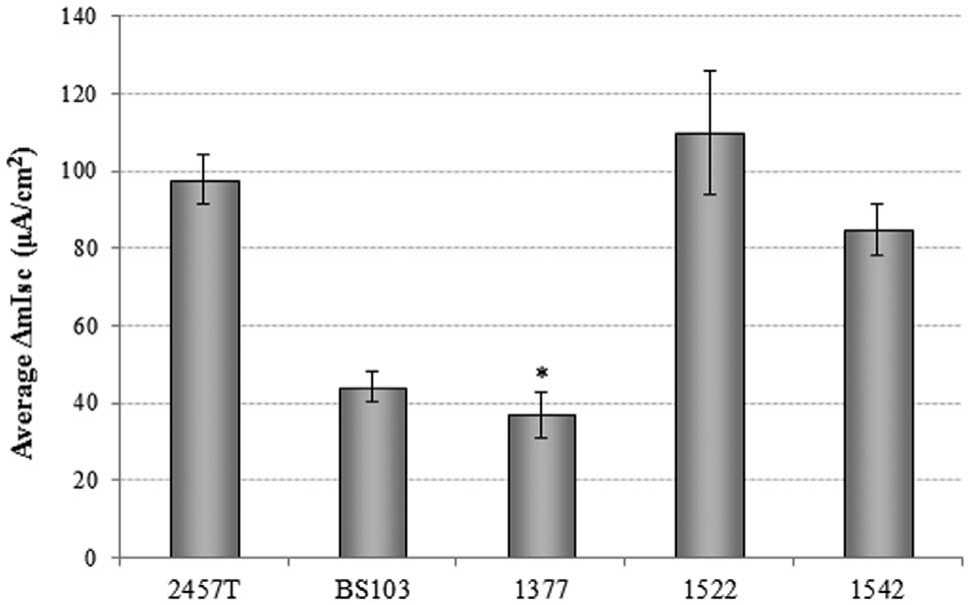
The *pic* and *set1AB* locus encodes the chromosomal toxins. Strain 1377 (Δ*pic*+Δ*set1AB*) had almost a 60% reduction in the change in short circuit current compared to wildtype 2457T. Complementation of the mutation in strain 1522 restores toxin activity to the same level as 2457T. In addition, addition of *pic* alone in strain 1542 restores ΔIsc to the same level as wildtype bacteria. The *p*-value difference between 2457T and 1377 is<0.001, as is the difference between 1377 and 1522 or 1542. There was no significant difference between 2457T, 1522, or 1542.

In order to identify the toxins encoded on the chromosome, we first analyzed a deletion mutant of the previously implicated *set1*A and *set1*B genes, which encode ShET1 (strain 1377). Studies have demonstrated that in *S. flexneri* 2a the *pic* gene is encoded on the opposite strand of DNA from that encoding the *set1*AB region [16]. To ensure that all potential enterotoxic activity was removed from this region, mutant 1377 had the entire *pic/set1*AB region deleted. Strain 1377 produced a change in Isc of approximately 40 µA/cm^2^ (Figure 4). Complementation of strain 1377 with the wildtype *pic/set1*AB locus replaced in strain 1522 restores enterotoxic activity to the same level as 2457T (Figure 4). The data therefore indicate that the chromosomal enterotoxic contribution arises from the ShET1/Pic region. Interestingly, when 1377 was complemented with *pic* alone in strain 1542, enterotoxic activity was restored to 2457T levels. In order to achieve complementation with *pic* alone, the locus was replaced with a cassette containing four point mutations that introduced stop codons in the *set1*A and *set1*B genes while maintaining the appropriate codons for *pic.* These data suggested that Pic is the enterotoxin producing the greater contribution. Expression of *set1*AB alone, however, was sufficient to induce a significant change in Isc [8], [9]. Therefore, we conclude that both Pic and ShET1 confer enterotoxic activity. Our data suggest that ShET1 and Pic are the chromosomally-encoded enterotoxins expressed under the culture conditions used in our experiments.

### Identification of the Genes Encoding the Virulence Plasmid Enterotoxins

**Figure 5:**
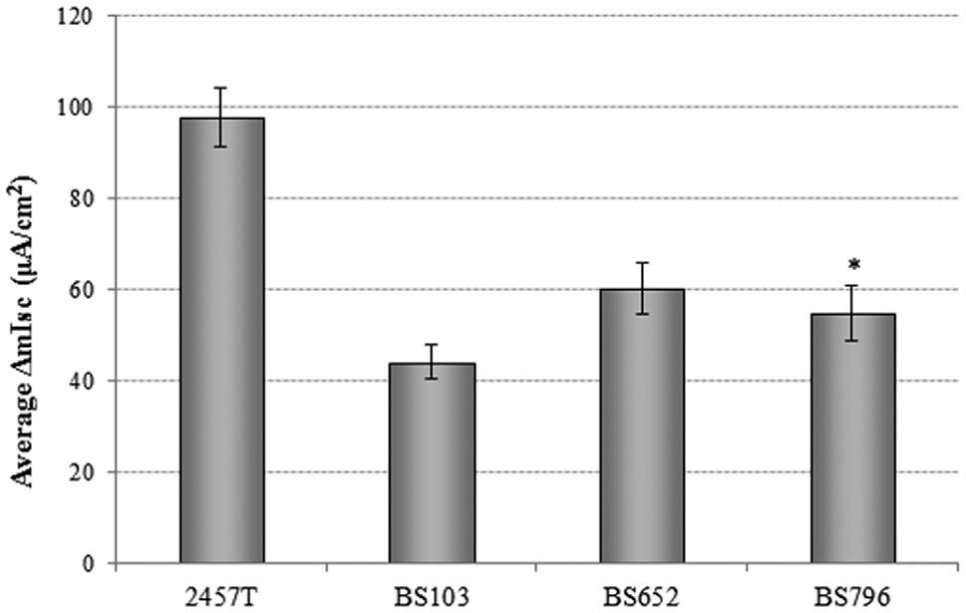
The type-III secretion system effector ShET2 (encoded by *sen* or *ospD3*) contributes to enterotoxin activity. A mutant that does not express ShET2 (BS796) causes the same change in short circuit current as the type-III secretion system mutant strain BS652. The *p*-value difference between 2457T and BS796 is<0.01. The data indicate that ShET2 is the type-III secretion system effector protein that contributes to enterotoxin activity.

Given that strain BS652 is a T3SS mutant and that ShET2 has been identified as a T3SS effector protein, we next analyzed strain BS796, which harbors a mutation in *sen* (also called *ospD3*), the gene encoding ShET2. As shown in figure 5, a mutation in ShET2 resulted in approximately the same ΔIsc as a mutant with a defective T3SS (strain BS652), suggesting that the enterotoxin secreted by the T3SS was in fact ShET2.

### Identification of Additional Enterotoxins Encoded by *Shigella flexneri*

**Figure 6:**
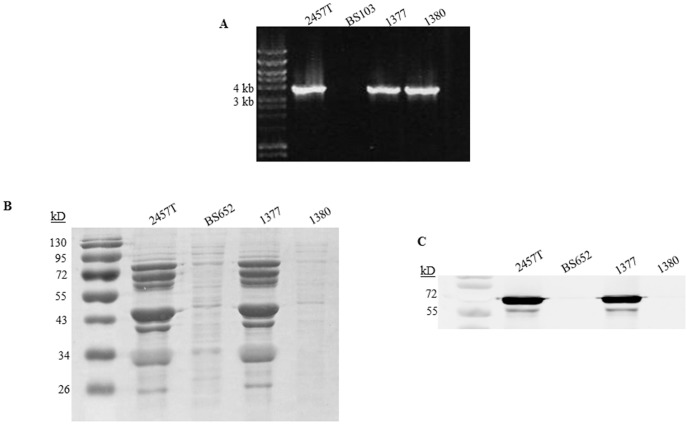
Strain 1380 has the virulence plasmid but a spontaneous mutation results in a defective T3SS. **A:** PCR analysis for the *ospC1* to *ospD3* coding region (3.7 kb) demonstrates presence of the virulence plasmid. The negative control is the plasmid-cured strain BS103. **B:** The Congo red secretion assay was performed, and the supernatants representing the secreted proteins were analyzed on an SDS-PAGE gel. The secretion profile of 2457T, BS652, 1377, and 1380 demonstrates that 1380 had a dysfunctional T3SS like the Δ*spa47* mutant (BS652). **C:** Western blot analysis of supernatants for IpaB. Secretion was only detected in the strains with a functional T3SS, strains 2457T and 1377.

The ΔIsc induced by strains 1380 and 1208S suggested that more enterotoxins were present in addition to Pic, ShET1, and ShET2. 1208S harbors a double mutation in ShET1 and ShET2. Strain 1380 is a spontaneous T3SS mutant of 1377 that was isolated by looking for white colonies on CR plates. PCR analysis for the *ospC1* to *ospD3* coding region (3.7 kb) revealed that 1380 still harbored the virulence plasmid (Figure 6A), and therefore was not a plasmid-cured strain of 1377. To verify that 1380 had a defective T3SS, a CR secretion assay was performed to ensure T3-secreted proteins were not present in supernatants. As shown in figure 6B, T3SS-specific secretion was significantly reduced in 1380, similar to the response in the *Δspa47* mutant (strain BS652). To confirm that secretion was T3SS-dependent, we performed Western blot analysis to detect the T3SS effector IpaB (Figure 6C); as expected, 1380 did not secrete IpaB. Therefore, we concluded that 1380 culture supernatants were deficient for Pic and ShET1 (due to the mutation), and ShET2 due to the lack of T3 effector secretion.

**Figure 7:**
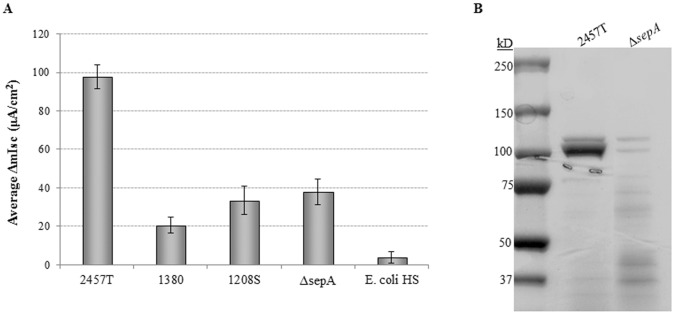
Additional *Shigella* proteins contribute to enterotoxin activity. **A:** Analysis of 1380 and 1208S verified that *S. flexneri* encodes enterotoxins in addition to Pic, ShET1, and ShET2. Analysis of a Δ*sepA* mutant demonstrated there was a significant decrease in the short circuit current, indicating that SepA contributed to enterotoxic activity. All 3 mutant strains have a significant increase in ΔIsc compared to *E. coli* HS with all *p*-values<0.01. **B:** Coomassie-stained protein gel of the secreted proteins obtained from the Δ*sepA* mutant after growth and concentration of cultural supernatants. SepA represents a large portion of the high molecular weight autotransporter proteins.

Strains 1208S and 1380 were analyzed using Ussing chambers to determine if some enterotoxic activity was retained despite removal or inadequate secretion of ShET1, Pic and, ShET2. Analysis of the secreted proteins from these strains demonstrated that enterotoxic activity was still present above *E. coli* HS levels (Figure 7A). These observations confirmed that *S. flexneri* encoded additional enterotoxins; and therefore, we proceeded to analyze a Δ*sepA* mutant generously provided by Dr. Beth McCormick. SepA is a Serine Protease Autotransporter of *Enterobacteriacae* (SPATE) that is encoded on the virulence plasmid [31]. This protein does not require the T3SS to be secreted, and therefore, served as a likely candidate to be the additional enterotoxin encoded on the virulence plasmid. The Δ*sepA* mutant elicited a ΔIsc significantly lower than wildtype (Figure 7A), indicating that SepA contributed to the enterotoxic activity.

## Discussion

The mechanism by which *Shigella* causes watery diarrhea is currently unknown. Since secretogenic activity is a central hallmark of early disease progression, the goal of this study was to expand previous work on *Shigella*-induced secretion through the validation of a mouse jejunum model using Ussing chambers. By analyzing several *S. flexneri* mutants, we were able to characterize the genomic determinants encoding toxins capable of increasing intestinal secretion signals. Previous studies in the field were aimed at characterizing ShET1 and ShET2 as *Shigella* enterotoxins but did not extend the study to address the presence of additional toxins. In the previous studies, the toxins were cloned and artificially expressed in *E. coli,* and the purified proteins were applied to rabbit jejunum mounted in Ussing chambers [4], [8], [9]. While this approach was important to demonstrate that toxin addition alone was sufficient to induce changes in Isc, the higher level of protein expressed and possible alterations from production in *E. coli* could affect the amount of short circuit change detected. By using the natural host, our studies with *S. flexneri* mutants allowed us to dissect the specific contribution of each enterotoxin previously described (ShET1 and ShET2) and of those newly discovered by our studies (Pic and SepA) in causing *Shigella* electrolyte secretion in the small intestine. The use of the natural host can also address potential interactions that occur between toxins and other secreted proteins.

A major advance, and a central aspect of our studies compared to previous reports, was the utilization of mouse tissue in the Ussing chamber experiments. We chose C57BL/6 mice as we will perform additional studies to address the roles of these toxins in knockout mice on the same genetic background lacking specific pattern recognition pathways or secretory pathways to gain more insights on the mechanism of action *Shigella*-induced secretory diarrhea. In addition, female mice have less of a tendency to fight, which is important since fighting can affect the immune system and other bodily systems. For these reasons this strain of mice was ideal for our studies. We have validated this model against the previously reported rabbit model in order to use mouse intestinal tissue as a reliable and efficient assay for our current and future studies. Our results demonstrate that mouse jejunum in the Ussing chamber system is as responsive to *Shigella* toxins as rabbit jejunum. Although several models of shigellosis have been developed, including the nonhuman primate model, the guinea pig keratoconjunctivitis model, and the pulmonary shigellosis model, the models do not adequately measure the watery diarrheal phase of infection [32], [33]. This study is the first to demonstrate secretogenic reactivity in an *ex vivo* mouse jejunum model to *Shigella* toxins.

The finding that the iron-depleted conditions were not necessary to study *Shigella* enterotoxic activities is an important aspect of this study. The iron-depleted medium that was initially utilized was based on the fact that these conditions were required to detect a change in Isc in studies with enteroinvasive *E. coli* [21]. Given the similarity between EIEC and *Shigella*, the iron-depleted conditions were continued into the *Shigella* studies [4], [8], [9]. In addition, supernatants isolated from a plasmid-cured strain of *S. flexneri* induced higher levels of water accumulation in the rabbit ileal loop model when the cultures were grown in the absence of iron [8]. However, another bacterial factor present in the culture supernatants, not ShET1, could have been affected by the iron limitation and enhanced water accumulation in the rabbit intestine. The data presented in figure 1 clearly demonstrate that iron-depletion is not required for Ussing chamber analysis of the *Shigella* enterotoxins. Whether iron limitation affects the efficacy of other *Shigella* proteins in the rabbit ileal loop analyses remains to be determined. Of course, use of other culture conditions could yield still more enterotoxins that may be produced *in vivo*.

Our mutant analysis strategy was instrumental in this study. We specifically chose to compare the plasmid-cured strain (BS103) and the Δ*spa47* mutant (BS652), a T3SS mutant that has been shown to be incapable of secreting T3SS effectors [24], [25]. Based on the secretion profile in figure 2, the autotransporters encoded on the virulence plasmid were still secreted in this mutant. In recent years three proteins belonging to the Serine Protease Autotransporter of *Enterobacteriacae* (SPATE) family have been identified in *S. flexneri*: SepA, SigA, and Pic. SepA is non-T3SS effector encoded on the virulence plasmid. Although the substrate(s) of SepA has not been identified, a recent study associated SepA with diarrheal cases in Mali [34]. SigA is encoded in the *she* pathogenicity island, and contributes to secretion in the rabbit ileal loop model [27]. Al-Hasani *et al.,* recently defined the structural protein fodrin as a target of SigA, implicating a role for this SPATE in cytoskeletal reorganization [35]. Finally, Pic is a mucinase that is encoded on the DNA strand directly opposite of the genes for ShET1. A recent study defined the substrate specificity of Pic, an immune system modulator [16]. Given the presence of the autotransporters in the culture supernatants and the association of these factors with diarrheal disease, we hypothesized that the SPATES could also contribute to enterotoxin activity. We therefore proceeded with our mutational analysis to determine if these SPATES had a role in inducing secretogenic activity.

Initially, analysis of BS103 and BS652 allowed us to determine if toxins were encoded on the chromosome and/or virulence plasmid. In addition, use of the Δ*spa47* mutant allowed us to demonstrate that the virulence plasmid harbored toxins that were T3-dependent and T3-independent since there was a significant increase in Isc compared to the plasmid-cured mutant (BS103) (figure 3). Based on our subtraction analysis, we conclude that three groups of toxins contribute to the increase in Isc: one group on the chromosome, one group on the virulence plasmid that requires the T3SS, and one group on the virulence plasmid that is secreted independently of the T3SS.

Further analysis indicated that the ShET1/Pic region is a major contributor of enterotoxin activity from the chromosome, which was confirmed by the analysis of the complementation of strain 1377 with ShET1-containing 1522 and ShET1-devoid 1542. Pic is transcribed on the opposite strand of DNA from the genes encoding ShET1, is only present in *S. flexneri*, and has been shown to target a wide range of leukocyte adhesion proteins [16]. How Pic contributes to secretogenic activity in our system and *in vivo* remains to be elucidated, and could function indirectly as an enterotoxin by affecting another protein. It is unclear in the previous literature whether or not the entire *pic* gene was present in the experiments in which the genes encoding ShET1 were cloned for expression and purification, but purified ShET1 has been shown to induce secretion in the rabbit ileal loop model [8], [9]. Our studies indicate that the majority of the enterotoxic activity present in *Shigella* strain 2457T is due to Pic.

Comparison of BS103 and the Δ*spa47* mutant indicated the presence of an additional factor encoded on the virulence plasmid that affected Isc. The Δ*ospD3* mutant analysis confirmed that ShET2 is the T3SS effector responsible for enterotoxin activity, which agrees with previous literature [4]. In fact, Nataro *et al.* hypothesized that ShET2 was a T3SS effector protein since a signal peptide could not be detected [4]. Analysis of strains 1208S and 1380 also indicated there were additional enterotoxins encoded by *S. flexneri*. We chose to analyze a Δ*sepA* mutant since it is an autotransporter encoded on the virulence plasmid that has been shown to affect fluid accumulation in rabbit ileal loops [31] and to contribute to diarrhea in enteroaggregative *E. coli* [34]. The Δ*sepA* mutant caused a significant drop in Isc compared to wildtype bacteria (Figure 7A). The data demonstrate that SepA has enterotoxic activity; however, there may be two reasons as to why the mutation caused such a dramatic drop in Isc compared to wildtype bacteria. First, analysis of the concentrated proteins from the mutant demonstrates that a significant amount of SepA is secreted into the cultures (Figure 7B). Second, SepA may target the same eukaryotic protein or pathway as another enterotoxin to enhance the overall toxin effect. Given the fact that in our culture conditions the Δ*spa47* mutant had an approximate 20% increase in Isc compared to BS103, and that strain 1380 (with only SepA present) had approximately 20% increase compared to the negative control *E. coli* HS, we believe that the SepA contribution is approximately 20% in our system. As with Pic, SepA could function indirectly as an enterotoxin by affecting another protein. Further characterization of SepA will determine the role of this autotransporter protein in our system and *in vivo*.

In conclusion, our study confirms the enterotoxin activity of ShET1 and ShET2 from previous publications, validates the use of mouse jejunum in Ussing chamber analyses as a novel, more versatile and high-throughput bioassay for *Shigella* enterotoxic activity, and identifies additional *Shigella* proteins (Pic and SepA) that contribute to enterotoxin activity. The results obtained in this study may assist future studies for identification of eukaryotic protein targets. The fact that we have yet to identify a toxin-null mutant indicates there may be additional enterotoxin(s) encoded by *S. flexneri* that contribute to the overall watery component of *Shigella*-induced diarrhea. Despite this possibility, we are confident that we have demonstrated that ShET1/Pic, ShET2, and SepA are the factors that contribute most prominently to the changes in Isc in mouse jejunum in the Ussing chambers.
