# Correction: Chromosomal and Plasmid-Encoded Factors of *Shigella flexneri* Induce Secretogenic Activity *Ex Vivo*

**DOI:** 10.1371/annotation/44400281-2456-4853-a89b-4dea4f9aa27b

**Published:** 2013-05-08

**Authors:** Christina S. Faherty, Jill M. Harper, Terez Shea-Donohue, Eileen M. Barry, James B. Kaper, Alessio Fasano, James P. Nataro

The name of the first author was missing a middle initial.

The correct name is: Christina S. Faherty

